# An Exploratory Study of Emotional Dysregulation Dimensions in Youth With Attention Deficit Hyperactivity Disorder and/or Bipolar Spectrum Disorders

**DOI:** 10.3389/fpsyt.2021.619037

**Published:** 2021-04-14

**Authors:** Gabriele Masi, Gianluca Sesso, Chiara Pfanner, Elena Valente, Agnese Molesti, Francesca Placini, Silvia Boldrini, Nina Loriaux, Flavia Drago, Anna Rita Montesanto, Simone Pisano, Annarita Milone

**Affiliations:** ^1^IRCCS Stella Maris, Scientific Institute of Child Neurology and Psychiatry, Pisa, Italy; ^2^Department of Clinical and Experimental Medicine, University of Pisa, Pisa, Italy; ^3^Department of Neuroscience, Santobono-Pausilipon Children Hospital, Naples, Italy; ^4^Department of Translational Medical Sciences, Federico II University, Naples, Italy

**Keywords:** emotional dysregulation, ADHD, bipolar disorder, children, adolescents

## Abstract

Emotional dysregulation (ED) is currently the most frequently used term to describe children with an impaired regulation of emotional states. Recent research studies speculate whether ED may be a neurodevelopmental disorder itself, a shared risk factor, or a common key feature of several psychiatric disorders, including, among others, attention deficit hyperactivity disorder (ADHD), and bipolar spectrum disorders (BSD). The association between ADHD and ED is one of the main reasons of misconceptions in the definition of boundaries between ADHD and BSD, leading to the frequent misdiagnosis of ADHD as BSD. Since ED is a multidimensional concept, a novel instrument—the Reactivity, Intensity, Polarity and Stability (RIPoSt) scale—was recently developed to assess the different dimensions of ED, which could help in detecting specific ED profiles in clinical youths. Our study included 154 patients, aged 13.8 ± 2.3 years, diagnosed with either ADHD, BSD, or comorbid condition, and a school-based sample of 40 healthy control (HC) adolescents, aged 12.5 ± 1.2 years. The RIPoSt scale and the Child Behavior Checklist were administered to both groups. Our results indicate that affective instability and negative emotionality subscales, as well as negative emotional dysregulation, are higher in BSD, both pure and comorbid with ADHD, while emotional impulsivity is higher in the comorbid condition and similar in the ADHD and BSD alone group; all clinical groups scored higher than HC. Conversely, positive emotionality is similar among clinical groups and within them and HC. Our findings also support the validity of the RIPoSt questionnaire, since the instrument proved to have good-to-excellent internal consistency, and strongly significant positive correlations were found with the CBCL-Dysregulation Profile, which is a commonly used, indirect measure of ED. Hence, the five subscales of the RIPoSt can be reliably used as an effective tool to study the emotional dysregulation in different clinical conditions, to help disentangle the complex relationship between ADHD and juvenile BSD and to provide clinicians with crucial evidence for better diagnostic characterization and therapeutic indications.

## Introduction

Children with an impaired regulation of emotional states, including mood lability and instability, severe irritability, low tolerance to frustration, temper outburst, and hyperarousal, have become a diagnostic challenge in the last two decades (1, 2). The core features of emotional impairment, with possible different combinations, are low threshold, excessive intensity, inappropriate expression, and slow normalization (1). This clinical picture does not completely fit any of the current nosological categories, including attention deficit hyperactivity disorder (ADHD), mood disorders (MD) such as bipolar spectrum disorders (BSD) or disruptive mood dysregulation disorder, and impulse control disorders such as oppositional defiant disorder (ODD)/conduct disorder (CD), although they may share features of all these domains. Different definitions of this condition have been proposed (3), but the term emotional dysregulation (ED) is currently the most frequently used.

More recent advances tend to interpret ED as a neurodevelopmental, early-onset disorder of the regulation of emotions, often associated to other psychiatric disorders, strongly related with comorbidity between internalizing and externalizing disorders, suggesting that it could be a shared risk factor for both kinds of disorder (4), or a common key factor in the development of later psychopathology (5–8).

Most if not all descriptions in children are focused on the association with ADHD (1, 9). At least 40% of subjects with ADHD present an associated ED (9–14), particularly in the combined presentation (15, 16), with strong continuity in adults with ADHD (17–19). Symptoms of ED significantly and negatively impact quality of life (11), social functioning (20), acceptability by peers (21), need for interventions (11), and higher rates of persistence of irritability and impulsivity up to adulthood (10). Moreover, ED has been shown to predict risky behaviors in adolescents with ADHD, such as, for instance, substance use and abuse, especially amphetamine and cannabinoids, other addictive behaviors, self-harm, and suicidality (22, 23). Finally, ED is a negative predictor of short-term response to methylphenidate monotherapy in drug-naïve youth with ADHD, especially of changes in hyperactive–impulsive symptoms, and thus should be systematically assessed in ADHD at baseline (24).

The association between ADHD and ED is one of the main reasons of misconceptions in the definition of boundaries between ADHD and BSD, leading to the frequent misdiagnosis of ADHD as BSD, or to an overinflated rate of comorbidity between ADHD and BSD. The issue of the boundaries between ADHD and BSD is still difficult to solve, given the partial overlap of symptoms, namely when ED is a prominent feature. Whether ED is an associated feature or a specifier of ADHD—which defines a specific subtype of the disorder—or even a core feature of the disorder—additional to hyperactivity/impulsivity and inattention—or, finally, a comorbidity, is still a matter of discussion (9). Recent advances in ED research revealed that it can also be a specific feature of youths with BSD (25), and unaffected relatives of BSD youth can still present subthreshold deficits in emotion regulation and processing (26).

One of the most troublesome issues in the assessment of dysregulated children is the availability of cost-effective and reliable diagnostic measures. To date, the Child Behavior Checklist (CBCL), one of the most used instruments for the assessment of developmental psychopathology (27), has been considered a possible diagnostic tool for identifying children with these features. The CBCL-Dysregulation Profile (CBCL-DP) is an indirect index of ED, characterized by simultaneously high values [above two standard deviations (SD)] in three syndrome scales (anxious/depressed, attention problems, and aggressive behavior). Interestingly, this index was initially thought to be more closely related to the pediatric BSD, and thus, it was named CBCL-Pediatric Bipolar Disorder profile (CBCL-PBD) (28). Further research has questioned this relationship (5, 29–31), supporting the notion that it may be of a measure of a wider dysregulation profile (DP), rather than a proxy for a single disorder (32). Consequently, longitudinal studies have highlighted that higher CBCL-DP scores in at-risk subjects predict the risk for substance use, suicidality, and poorer overall functioning (5). Similarly, higher scores of DP in ODD patients predict a greater risk for ADHD and mood disorder in adolescence (33), while higher scores in ADHD patients predict impaired psychosocial functioning, psychiatric hospitalizations, and subsequent diagnoses of CD and BSD at the follow-up (34). In other words, research clearly suggests that ED, as indirectly assessed with an empirically derived measure (CBCL-DP), has high clinical relevance in different kinds of samples.

Although ED is a multidimensional concept, including emotional reactivity and impulsivity, affective intensity and polarity—both positive or elated and negative or irritable—and behavioral self-control, CBCL does not allow clinicians to disentangle these different components, which may be different in different subjects. Assessing all these components may need different measures, which are currently unavailable in youth (35, 36). However, a recently developed instrument to assess these different dimensions is the Reactivity, Intensity, Polarity, and Stability (RIPoSt) scale (37).

Starting from 60 items concerning reactivity, intensity, polarity of emotional responses, and affective stability, a first validation in both clinical and non-clinical adult subjects led to a 40-item version with four scales (38). The four scales are the following: affective instability (AI), with 12 items exploring the presence of a cyclic pattern of sudden mood shifts between positive and negative polarity; emotional impulsivity (EI), with 8 items on the over-reactivity to negative or frustrating stimuli and the inability to inhibit impulsive behavioral responses; negative emotionality (NE), with 10 items evaluating the propensity for experiencing more often and more easily strong negative feelings, such as sadness, worry, anxiety and dissatisfaction; and positive emotionality (PE), with 10 items exploring the tendency to experience more often and more easily strong positive feelings, such as euphoria, joy, enthusiasm, and exuberance. The first three subscales also sum up to a negative ED (NED) scale, totally including 30 items. Measures of reliability (test–retest *r* = 0.71–0.84) and internal consistency (Cronbach's α = 0.72–0.95) were high, and concurrent validity was also supported by correlations with the brief TEMPS-M subscales (39). Discriminant validity was finally significant (*p* <0.001) since cyclothymic and ADHD patients exhibited higher scores than non-clinical controls for each subscale, except for PE.

In the present exploratory study, we employed, for the first time, the 40-item version of the RIPoSt questionnaire in a clinical and non-clinical sample of youths, providing initial psychometric assessment and thoroughly examining ED profiles in a sample of ADHD and/or BSD patients, in order to detect possible specificities. Our main hypotheses are that emotional regulation is more impaired in the comorbid condition (ADHD + BSD) than in ADHD or BSD alone patients and that all clinical groups score higher than a control group of healthy adolescents in all subscales of the questionnaire. We lack specific *a priori* hypotheses on each single dimension of the construct, since no previous clinical study applied the RIPoSt questionnaire in youths. Nonetheless, according to the theoretical model proposed by Banaschewski et al. (40) and Petrovic and Castellanos (41), we may only hypothesize a selective increase in EI scores in ADHD patients, both pure and comorbid with BSD, unless this specific subscale reflects the high sensitivity to emotionally salient stimuli with reduced self-control and behavioral inhibition described by the model.

## Materials and Methods

### Recruitment and Diagnostic Procedures

Our study included 154 participants (104 males and 50 females, age range 9–18 years, mean age 13.8 ± 2.3 years) recruited in our third-level Department of Child and Adolescent Psychiatry and Psychopharmacology from 2017 to 2020 (clinical group; CG). Inclusion criteria were diagnoses of ADHD, BSD, or both, made according to the Diagnostic and Statistical Manual of Mental Disorders—fifth edition (DSM-5) (42), based on medical history, clinical observations, and a semistructured interview, the Kiddie Schedule for Affective Disorders and Schizophrenia—Present and Lifetime version (K-SADS-PL) (43), administered by trained child psychiatrists to both patients and parents.

Exclusion criteria for the CG were as follows: older than 18 years old or younger than 9 years old; presence of comorbid intellectual disability, as detected through formal psychometric assessment (either the Full-Scale Intelligence Quotient or the General Ability Index below 85 at the WISC-IV); and presence of comorbid autism spectrum disorders, schizophrenia spectrum, and other psychotic disorders.

Three clinical subgroups were identified in the CG: the ADHD group (namely, without comorbid BSD), consisting of 72 subjects (62 males and 10 females, mean age 12.9 ± 2.2 years); the BSD group (namely, without comorbid ADHD), consisting of 53 subjects (18 males and 35 females, mean age 14.9 ± 1.8 years); and the comorbid ADHD + BSD group, consisting of 29 subjects (24 boys and 5 females, mean age 13.8 ± 2.4 years).

A school sample of 40 healthy control adolescents (HC group) (8 boys and 32 females, age range 9–18 years old, mean age 12.5 ± 1.2 years) was recruited on a voluntary basis upon engagement of a nearby junior high school in Pisa. Exclusion criteria for the HC group were as follows: older than 18 years old or younger than 9 years old, presence of intellectual disability, and presence of any psychiatric disorder.

All participants and parents were informed about assessment instruments, and there was voluntary participation in the study after written informed consent was obtained for assessment procedures from the parents of all children. The institutional review board of our hospital approved the study.

### Measures

A clinical questionnaire, the Child Behavior Checklist, was used in the both CG and HC samples to support clinical assessment and diagnostic procedures. The Child Behavior Checklist for ages 6–18 years (CBCL-6/18) (27, 44) is a 118-item scale, completed by parents or caregivers, with eight different syndromes scales, a total problem score, and two broad-band scores designated as internalizing problems and externalizing problems. In the current study, emotional dysregulation was assessed based on the CBCL-DP, using the sum of *t* scores of the following subscales, anxious/depression, attention problems, and aggressive behaviors. The reliability coefficients (Cronbach's α) were 0.82, 0.81, and 0.82, respectively.

CG and HC were also assessed by means of the Italian 40-item version of the Reactivity, Intensity, Polarity and Stability (RIPoSt-40) questionnaire (37, 38), a self-rated measure of emotional dysregulation. The RIPoSt-40 has been recently validated in an adult Italian sample of 174 cyclothymic and/or ADHD patients and 396 non-clinical subjects. The 40 items are unequally distributed across four subscales, respectively identified as measures of AI, EI, NE, and PE; the first three subscales also sum up to a NED score which includes 30 items. The instrument showed generally high test–retest reliability (*r* = 0.71–0.84) and good-to-excellent internal consistency (Cronbach's α = 0.72–0.95). Concurrent and discriminant validity were also demonstrated to be significant. Thus, the RIPoSt-40 questionnaire proved to be a valid, reliable, and useful tool to assess emotional dysregulation, both in clinical and non-clinical contexts.

### Statistics

Statistical analyses were performed by means of MATLAB® and RStudio® software. For each clinical variable with continuous distribution, outliers were defined as observations lying outside the range between (first quartile − 2 ^*^ interquartile range) and (third quartile + 2 ^*^ interquartile range) and removed. Cronbach's alphas were computed as measures of internal consistency of each subscale of the RIPoSt-40 questionnaire. The χ^2^ test was used to detect significant differences (*p* <0.05) between the three clinical groups and the HC group in the distributions of demographic and clinical nominal categorical variables, such as gender and clinical comorbidities. When more than 20% of observations had expected frequencies <5, Fisher's exact test was performed. Analyses of covariance (ANCOVA) were conducted to assess significant differences (*p* <0.05) between group means in the demographic and clinical variables with continuous distribution, such as subscale scores of the RIPoSt-40 questionnaire while controlling for gender as covariate. A Tukey *post hoc* test was used whenever ANCOVA led to a statistically significant result in order to identify significant comparisons between couples of groups.

Pearson's linear correlation coefficients were estimated to detect significant relationships of the RIPoSt-40 questionnaire subscales with each other and between these and the CBCL-6/18 subscales in the CG and HC group. The Bonferroni correction method for multiple comparisons was applied after assessing significant differences at a traditional significance level of 5%. Finally, linear multivariate regression models were applied to identify statistically significant associations between the RIPoSt-40 questionnaire subscales and the presence of psychiatric comorbidities, notably anxiety disorders and disruptive behavior disorders, while controlling for the principal diagnoses (ADHD and BSD) as covariates.

## Results

Our sample included 194 participants, of which 154 were in the CG (72 ADHD, 53 BSD, and 29 ADHD + BSD) and 40 in the HC group. Demographic and clinical characteristics of the four groups are reported in Table 1. As shown, gender and age were significantly different among the groups; *post hoc* comparisons are detailed in the table legend. Clinical comorbidities also significantly differed, with the BSD group exhibiting the greatest mean number of comorbid psychiatric conditions, followed by the ADHD + BSD, and then by the ADHD. Specific comorbidities, according to DSM-5, are listed in Table 1.

**Table 1:** Demographic and clinical characteristics of the sample.

**Total = 194**	**Group 1 ADHD**	**Group 2 BSD**	**Group 3 ADHD + BSD**	**Group 4 HC**	***p***
*N*	72	53	29	40	–
Males, *N* (%)	62 (86.1)	18 (34.0)	24 (82.8)	8 (20)	<0.001***
Age, *M* (SD)	12.9 (2.2)	14.9 (1.8)	13.8 (2.4)	12.5 (1.2)	<0.001***
Comorbidities, *M* (SD)	0.8 (1.0)	2.7 (1.0)	1.8 (1.5)	0 (0)	<0.001***
Single AD, *N* (%)	9 (12.5)	13 (24.5)	6 (20.7)	0 (0)	<0.001***
Multiple AD, *N* (%)	7 (9.7)	20 (37.7)	7 (24.1)	0 (0)	
OCD, *N* (%)	2 (2.8)	6 (11.3)	2 (6.9)	0 (0)	0.017*
Tics, *N* (%)	5 (6.9)	1 (1.9)	3 (10.3)	0 (0)	0.134
ODD, *N* (%)	21 (29.2)	18 (34.0)	16 (55.2)	0 (0)	<0.001***
CD, *N* (%)	3 (4.2)	11 (20.8)	4 (13.8)	0 (0)	<0.001***
Eating disorders, *N* (%)	1 (1.4)	6 (11.3)	0 (0)	0 (0)	0.003**

Post hoc comparisons: age: group 1–group 2: p <0.001 <0.001***; group 1–group 3: p = 0.208; group 1–group 4: p = 0.703; group 2–group 3: p = 0.061; group 2–group 4: p <0.001 <0.001***; group 3–group 4: p = 0.044*. Comorbidities: group 1–group 2: p <0.001 <0.001***; group 1–group 3: p <0.001 <0.001***; group 1–group 4: p <0.001 <0.001**; group 2–group 3: p = 0.005**; group 2–group 4: p <0.001 <0.001***; group 3–group 4: p <0.001 <0.001***. AD, anxiety disorder; ADHD, attention deficit hyperactivity disorder; BSD, bipolar spectrum disorder; CD, conduct disorder; HC, healthy controls; M, mean; N, number; OCD, obsessive–compulsive disorder; ODD, oppositional defiant disorder; SD, standard deviation. *p <0.05; **p <0.01; ***p <0.001.

Internal consistency of the RIPoSt-40 questionnaire was initially assessed by computing Cronbach's α coefficients for each subscale. Cronbach's coefficients were generally high for most subscales (AI: α = 0.896; EI: α = 0.870; NE: α = 0.864; AI: α = 0.896), except for PE, whose internal consistency was still good (PE: α = 0.814). An excellent reliability value was identified for the NED subscale (NED: α = 0.946).

**Figure 1:**
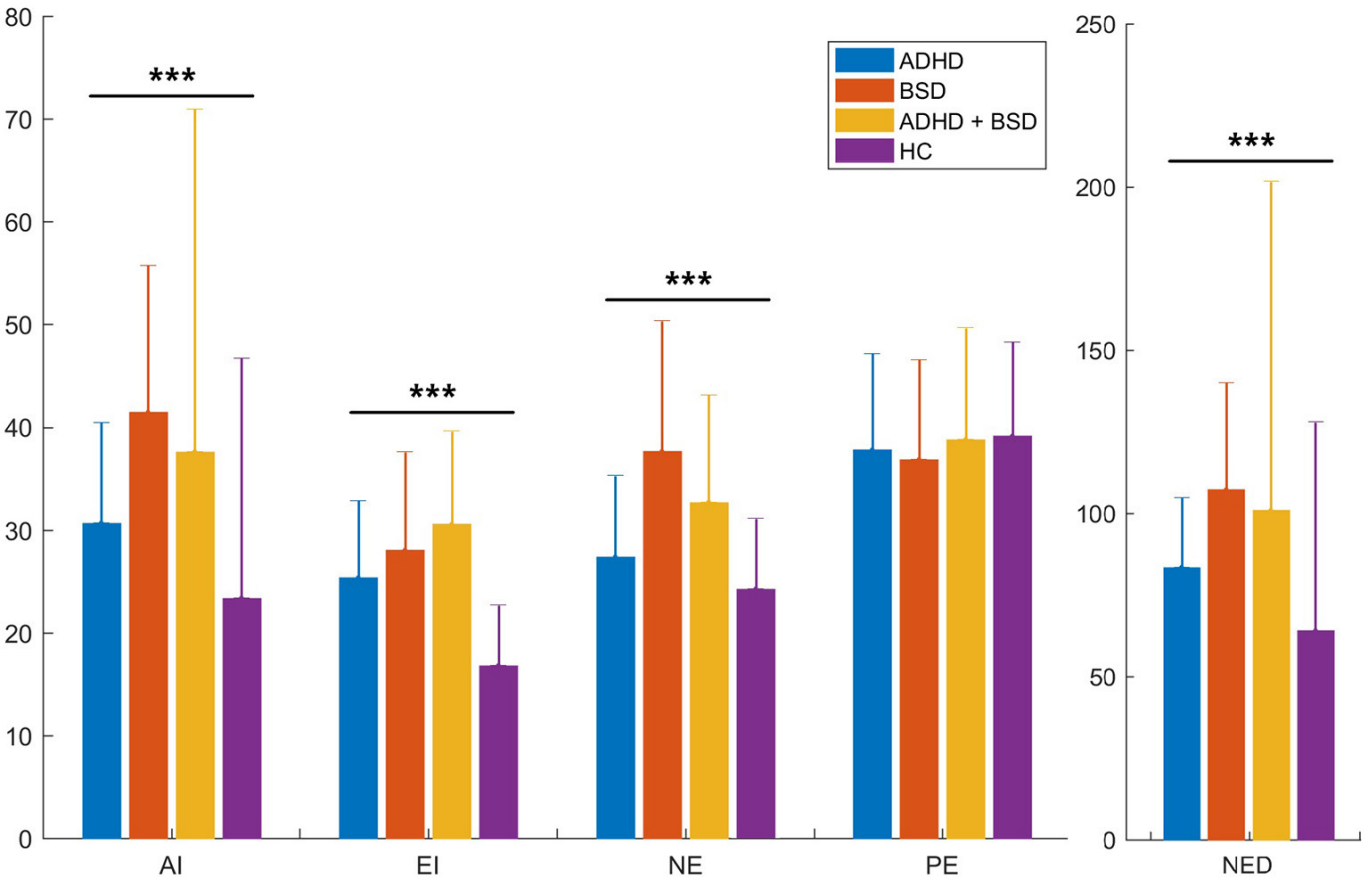
RIPoSt-40 subscales: comparisons between clinical and non-clinical groups. Scores obtained by the four groups in our sample, namely the three clinical groups and the control group, in the five RIPoSt-40 questionnaire subscales—AI, EI, NE, PE, and NED—are here illustrated. Scores are compared between ADHD patients (blue bars), BSD patients (red bars), comorbid ADHD + BSD patients (yellow bars), and HC individuals (purple bars). Graphs represent means with standard deviation bars. ADHD, attention deficit hyperactivity disorder; AI, affective instability; BSD, bipolar spectrum disorder; EI, emotional impulsivity; HC, healthy controls; NE, negative emotionality; NED, negative emotional dysregulation; PE, positive emotionality; RIPoSt-40, Reactivity, Intensity, Polarity and Stability questionnaire. ***p < 0.001.

We then compared the RIPoSt-40 subscale scores between the three CG and the HC through ANCOVAs, while correcting for gender distributions as covariate. Age was also initially assessed through a linear multivariate model, though displaying no significant effects on any of the questionnaire subscales and not altering the effect of the other variables of the model; thus, we decided to remove it from the analyses. As shown in Table 2 and Figure 1, the AI, EI, NE, and NED subscales demonstrated highly significant differences among the groups, while the analysis revealed no significant effect of diagnosis or gender on the PE subscale. *Post hoc* comparisons are detailed in the table legend. Notably, the BSD and ADHD + BSD groups scored the highest in the AI, NE, and NED subscales, without significant differences between the groups, and the ADHD group presented significantly lower scores in the three scales, but higher than the HC group. As for the EI subscale, the ADHD + BSD group scored the highest, followed by the BSD and the ADHD groups, which did not differ significantly between them, and finally the HC group, with significantly lower scores. *Post hoc* comparisons between males and females in the RIPoSt-40 questionnaire subscales revealed highly significant gender-related differences for the AI, NE, and NED subscales, with females scoring higher than males (data not shown).

**Table 2:** RIPoSt-40 subscales: comparisons among the PAT and HC groups.

**Total = 194**	**Group 1 ADHD**	**Group 2 BSD**	**Group 3 ADHD + BSD**	**Group 4 HC**	***p***
*N*	72	49	28	38	–
RIPoSt-40 AI, *M* (SD)	30.7 (9.8)	41.4 (14.3)	37.6 (12.2)	23.4 (9.1)	<0.001***
RIPoSt-40 EI, *M* (SD)	25.3 (7.5)	28.0 (9.6)	30.5 (9.1)	16.8 (5.9)	<0.001***
RIPoSt-40 NE, *M* (SD)	27.4 (7.9)	37.6 (12.7)	32.7 (10.5)	23.8 (7.3)	<0.001***
RIPoSt-40 PE, *M* (SD)	37.8 (9.4)	36.8 (9.7)	38.8 (10.9)	39.1 (9.2)	0.702
RIPoSt-40 NED, *M* (SD)	83.4 (21.5)	107.1 (33.0)	100.8 (28.3)	64.0 (20.4)	<0.001***

Post hoc comparisons: AI: group 1–group 2: p <0.001 <0.001***; group 1–group 3: p = 0.027*; group 1–group 4: p = 0.007**; group 2–group 3: p = 0.464; group 2–group 4: p <0.001 <0.001***; group 3–group 4: p <0.001 <0.001***; males < females: p = 0.001**. EI: group 1–group 2: p = 0.279; group 1–group 3: p = 0.022*; group 1–group 4: p <0.001 <0.001**; group 2–group 3: p = 0.555; group 2–group 4: p <0.001 <0.001***; group 3–group 4: p <0.001 <0.001***; males < females: p = 0.087. NE: group 1–group 2: p <0.001 <0.001***; group 1–group 3: p = 0.056; group 1–group 4: p = 0.244; group 2–group 3: p = 0.119; group 2–group 4: p <0.001 <0.001***; group 3–group 4: p = 0.001**; males < females: p <0.001 <0.001***. PE: group 1–group 2: p = 0.952; group 1–group 3: p = 0.968; group 1–group 4: p = 0.903; group 2–group 3: p = 0.834; group 2–group 4: p = 0.698; group 3–group 4: p = 0.999; males < females: p = 0.409. NED: group 1–group 2: p <0.001 <0.001***; group 1–group 3: p = 0.011*; group 1–group 4: p <0.001 <0.001***; group 2–group 3: p = 0.719; group 2–group 4: p <0.001 <0.001***; group 3–group 4: p <0.001 <0.001***; males < females: p = 0.001**. AI, affective instability; ADHD, attention deficit hyperactivity disorder; BSD, bipolar spectrum disorder; EI, emotional impulsivity; HC, healthy controls; M, mean; N, number; NE, negative emotionality; NED, negative emotional dysregulation; PE, positive emotionality; RIPoSt-40, 40-item Reactivity, Intensity, Polarity and Stability questionnaire; SD, standard deviation. *p < 0.05; **p <0.01; ***p < 0.001.

As shown in Table 3, the AI, EI, and NE subscales were all highly significantly positively correlated in the whole sample, with coefficients *r* ranging between 0.660 for the correlation between AI and EI and 0.829 for the correlation between AI and NE. The PE subscale was also positively correlated, though less significantly, with the AI, EI, and NE subscales, with coefficients *r* between 0.184 for the correlation with NE and 0.227 for the correlation with EI. Significantly positive correlations were finally identified between the NED and other subscales of the questionnaire.

**Table 3:** RIPoSt-40 subscale internal correlations.

	**RIPoSt-40 AI**	**RIPoSt-40 EI**	**RIPoSt-40 NE**	**RIPoSt-40 PE**	**RIPoSt-40 NED**
RIPoSt-40 AI	*r* = 1	*r* = 0.660	*r* = 0.829	*r* = 0.184	*r* = 0.938
	*p* = 1	*p* <0.001 <0.001***	*p* <0.001 <0.001***	*p* = 0.012*	*p* <0.001 <0.001***
RIPoSt-40 EI	*r* = 0.660	*r* = 1	*r* = 0.667	*r* = 0.227	*r* = 0.835
	*p* <0.001 <0.001***	*p* = 1	*p* <0.001 <0.001***	*p* = 0.002**	*p* <0.001 <0.001***
RIPoSt-40 NE	*r* = 0.829	*r* = 0.667	*r* = 1	*r* = 0.158	*r* = 0.928
	*p* <0.001 <0.001***	*p* <0.001 <0.001***	*p* = 1	*p* = 0.031*	*p* <0.001 <0.001***
RIPoSt-40 PE	*r* = 0.184	*r* = 0.227	*r* = 0.158	*r* = 1	*r* = 0.207
	*p* = 0.012*	*p* = 0.002**	*p* = 0.031*	*p* = 1	*p* = 0.005*
RIPoSt-40	*r* = 0.938	*r* = 0.835	*r* = 0.928	*r* = 0.207	*r* = 1
NED	*p* <0.001 <0.001***	*p* <0.001 <0.001***	*p* <0.001 <0.001***	*p* = 0.005*	*p* = 1

AI, affective instability; EI, emotional impulsivity; NE, negative emotionality; NED, negative emotional dysregulation; PE, positive emotionality; RIPoSt-40, 40-item Reactivity Intensity Polarity Stability questionnaire. *p < 0.05; **p < 0.01; ***p < 0.001.

We also estimated linear correlation coefficients to detect significant relationships between the RIPoSt-40 and the CBCL-6/18 subscales. The AI, EI, NE, and NED subscales of the RIPoSt-40 questionnaire were highly significantly positively correlated with all syndromes and problems subscales and most DSM-oriented diagnostic category subscales, while the only significant negative relationships of the PE subscale were identified with the anxious/depressed, the withdrawn/depressed, and the affective problems subscales. Notably, the dysregulation profile index of the CBCL-6/18 was highly positively associated with the AI, EI, NE, and NED subscales, while no significant correlation was detected with the PE subscale. Correlation coefficients and statistics are detailed in Tables 4A,B.

**Table 4:** RIPoSt-40 and CBCL-6/18 subscale correlations.

	**RIPoSt-40 AI**	**RIPoSt-40 EI**	**RIPoSt-40 NE**	**RIPoSt-40 PE**	**RIPoSt-40 NED**
**(A) Coefficients** ***r***					
CBCL AD	0.440	0.457	0.492	−0.171	0.502
CBCL WD	0.431	0.341	0.441	−0.240	0.445
CBCL SomP	0.382	0.249	0.427	−0.014	0.391
CBCL SocP	0.448	0.473	0.462	0.012	0.499
CBCL TP	0.381	0.439	0.386	−0.061	0.433
CBCL AP	0.322	0.430	0.279	−0.030	0.367
CBCL RBB	0.368	0.474	0.314	0.045	0.412
CBCL AB	0.331	0.482	0.322	0.042	0.402
CBCL DPI	0.428	0.530	0.426	−0.066	0.495
CBCL Int	0.463	0.480	0.495	−0.122	0.520
CBCL Ext	0.397	0.546	0.367	0.028	0.466
CBCL Tot	0.437	0.544	0.436	−0.049	0.507
CBCL Aff	0.310	0.298	0.323	−0.213	0.343
CBCL Anx	0.167	0.250	0.276	−0.180	0.250
CBCL Som	0.189	0.078	0.302	0.0172	0.219
CBCL ADHD	−0.048	0.208	−0.058	0.020	0.018
CBCL ODP	0.061	0.286	0.107	0.025	0.150
CBCL CP	0.098	0.338	0.054	0.040	0.161
**(B)** ***p*** **values**					
CBCL AD	<0.001***	<0.001***	<0.001***	0.045*	<0.001***
CBCL WD	<0.001***	<0.001***	<0.001***	0.004**	<0.001***
CBCL SC	<0.001***	0.003**	<0.001***	0.862	<0.001***
CBCL SP	<0.001***	<0.001***	<0.001***	0.881	<0.001***
CBCL TP	<0.001***	<0.001***	<0.001***	0.477	<0.001***
CBCL AP	<0.001***	<0.001***	<0.001***	0.726	<0.001***
CBCL RBB	<0.001***	<0.001***	<0.001***	0.600	<0.001***
CBCL AB	<0.001***	<0.001***	<0.001***	0.619	<0.001***
CBCL DPI	<0.001***	<0.001***	<0.001***	0.445	<0.001***
CBCL Int	<0.001***	<0.001***	<0.001***	0.156	<0.001***
CBCL Ext	<0.001***	<0.001***	<0.001***	0.745	<0.001***
CBCL Tot	<0.001***	<0.001***	<0.001***	0.569	<0.001***
CBCL Aff	0.001**	0.002**	0.001**	0.034*	<0.001***
CBCL Anx	0.096	0.012*	0.005**	0.074	0.012*
CBCL Som	0.061	0.441	0.002**	0.865	0.029*
CBCL ADHD	0.633	0.038*	0.566	0.840	0.858
CBCL ODP	0.547	0.004**	0.290	0.803	0.135
CBCL CP	0.331	<0.001***	0.590	0.688	0.109

ADHD, attention deficit hyperactivity disorder; AB, aggressive behavior; AD, anxious/depressed; Aff, affective problems; AI, affective instability; Anx, anxiety problems; AT, attention problems; CBCL, Child Behavior Checklist; CP, conduct problems; DPI, dysregulation profile index; EI, emotional impulsivity; Ext, externalizing problems; Int, internalizing problems; NE, negative emotionality; NED, negative emotional dysregulation; ODP, oppositional defiant problems; PE, positive emotionality; RBB, rule-breaking behavior; RIPoSt-40, 40-item Reactivity, Intensity, Polarity and Stability questionnaire; SC, somatic complaints; Som, somatic problems; SP, social problems; Tot, total problems; TP, thought problems; WD, withdrawn/depressed. *p < 0.05; **p < 0.01; ***p < 0.001.

Five linear multivariate regression models were finally applied to identify statistical associations between the subscales of the RIPoSt-40 questionnaire, as dependent variables, and the presence of psychiatric comorbidities [i.e., single and/or multiple anxiety disorder (AD) and ODD and/or CD], as independent variables, while controlling for the principal diagnoses (ADHD and BSD). As displayed in Tables 5A–E, significant positive associations were found between the AI, NE, and NED subscales and both BSD and multiple AD. Moreover, EI was significantly positively associated with both ADHD and BSD, while PE displayed no significant associations. Neither the presence of a single AD nor that of ODD/CD was significantly associated with any of the RIPoSt-40 subscales.

**Table 5:** Linear regression models with clinical comorbidities.

	**β**	**SE**	***t* value**	***p***
**(A) RIPoSt-40 AI**				
Intercept	26.099	1.626	16.051	<0.001***
ADHD	1.527	1.863	0.820	0.414
BSD	10.001	2.348	4.259	<0.001***
Single AD	4.147	2.653	1.563	0.120
Multiple AD	6.756	2.620	2.578	0.011*
ODD/CD	1.724	2.094	0.823	0.412
**(B) RIPoSt-40 EI**				
Intercept	18.105	1.134	15.969	<0.001***
ADHD	5.720	1.299	4.404	<0.001***
BSD	7.088	1.637	4.329	<0.001***
Single AD	2.265	1.850	1.224	0.223
Multiple AD	2.598	1.827	1.422	0.157
ODD/CD	0.426	1.460	0.292	0.771
**(C) RIPoSt-40 NE**				
Intercept	26.081	1.342	19.436	<0.001***
ADHD	−0.232	1.537	−0.151	0.880
BSD	9.137	1.938	4.714	<0.001***
Single AD	3.178	2.190	1.450	0.149
Multiple AD	5.672	2.163	2.623	0.006**
ODD/CD	−2.127	1.729	−1.231	0.220
**(D) RIPoSt-40 PE**				
Intercept	38.513	1.373	28.044	<0.001***
ADHD	−0.463	1.573	−0.294	0.769
BSD	−0.340	1.984	−0.172	0.864
Single AD	3.419	2.241	1.526	0.129
Multiple AD	−0.380	2.213	−0.172	0.864
ODD/CD	−0.624	1.769	−0.353	0.725
**(E) RIPoSt-40 NED**				
Intercept	70.285	3.629	19.369	<0.001***
ADHD	7.015	4.157	1.687	0.093
BSD	26.226	5.241	5.004	<0.001***
Single AD	9.587	5.921	1.619	0.107
Multiple AD	15.026	5.848	2.569	0.011*
ODD/CD	0.023	4.674	0.005	0.996

ADHD, attention deficit hyperactivity disorder; AD, anxiety disorders; AI, affective instability; BSD, bipolar spectrum disorders; CD, conduct disorder; EI, emotional impulsivity; NE, negative emotionality; NED, negative emotional dysregulation; ODD, oppositional defiant disorder; PE, positive emotionality; RIPoSt-40, 40-item Reactivity, Intensity, Polarity and Stability questionnaire; SE, standard error. *p < 0.05; **p < 0.01; ***p < 0.001.

## Discussion

This is the first study aimed to explore ED in a clinical sample of children and adolescents using a specific measure, the RIPoSt questionnaire, which includes four dimensions of dysregulation, that is affective instability, emotional impulsivity, negative emotionality, positive emotionality, and the negative emotional dysregulation derived from the sum of the first three dimensions. The first aim of our study was to explore the different dimensions of ED in youth with ADHD, BSD, and the two comorbid conditions. The secondary aims of the study were to preliminarily explore the psychometric characteristics of the RIPoSt questionnaire and to compare this measure with a well-established dimensional measure of psychopathology in youth, the CBCL-6/18, and more specifically with the CBCL-Dysregulation Profile, derived from the three symptom scales of the instrument.

The boundaries between ADHD and BSD raised a controversy in the literature, given the partial overlap of symptoms, such as hyperactivity, impulsivity/aggressiveness, and distractibility, particularly when ED is associated. Indeed, when this latter is prominent, inflated rates of comorbidity between the two disorders have been reported in the literature (45). The greater awareness of ED in ADHD individuals (1, 14) has contributed to a better comprehension of the relationship between ADHD and BSD, but the lack of reliable and sensitive measures of ED significantly limited this exploration. Thus, the RIPoSt questionnaire may represent a possible new tool for exploring different dimensions of ED in ADHD, BSD, and the comorbid condition, compared with heathy controls, which helps to better understand the relationship between the two disorders and to finely disentangle the disorders, highlighting possible targets for a well-adjusted intervention.

Our findings indicate that AI and NE, as well as the combined NED scale, are mostly related to the BSD, both pure and with ADHD, and can reliably differentiate these conditions from pure ADHD. Similarly, these three dimensions are able to discriminate the dysregulated profile of ADHD youth compared with the heathy controls. A more nuanced difference was shown with emotional impulsivity, which was found to be similar in ADHD and BSD alone and higher in the comorbid condition, and notably, all clinical groups exhibited higher scores than the healthy controls.

On the other hand, PE is unable to differentiate clinical and healthy groups and seems a sort of temperamental dimension, which can be found in both patients and healthy individuals, without a significant impairing effect. Moreover, it seems more difficult to be detected, at least compared with NE, and would thus require larger sample size to achieve statistical significance (38). Also, differences among groups in PE may be more qualitative than quantitative, but even more reactive and transitory in clinical samples, and/or with different behavioral correlates. Further studies are hence needed to support the clinical utility of the PE subscale.

A comparison between these results and those reported in a parallel study, conducted on adult patients explored with the same diagnostic tool (38), is highly informative, given the strong consistencies in the findings, with remarkable implications in a developmental perspective. In Brancati et al. (38), the RIPoSt questionnaire was administered to two clinical samples, namely cyclothymic and ADHD patients, along with a community-based sample of healthy controls. Consistent with our data, AI, NE, and NED lead to overlapping scores in cyclothymic and ADHD patients, and both groups scored higher than healthy controls, while PE failed to discriminate clinical patients and healthy individuals. Noteworthy, adult ADHD scored higher than both cyclothymic and healthy individuals in the EI subscale, suggesting that this dimension would be more likely related to the hyperactive–impulsive trait of ADHD rather than to the affective instability of BSD. On the contrary, in our youth, ADHD and BSD exhibited similar scores in EI, and only the comorbid condition was associated with higher scores. A possible explanation of this phenomenon may be related to the developmental divergences between juvenile and adult BSD, since among youth, impulsivity, both emotional and behavioral, is considered as a marker of earlier-onset juvenile BSD, which makes this condition more similar to ADHD (46). Conversely, adult BSD is less impulsive and more affective, while impulsivity of ADHD adult patients is much more prominent.

Our findings also provide a preliminary support to the construct and concurrent validity of the 40-item version of the RIPoSt questionnaire to assess ED. Indeed, the instrument proved to have good-to-excellent internal consistency in both clinical and non-clinical samples. Cronbach's coefficients were high for all subscales and for their combination in the NED subscale, while they were lesser, though still good, for the PE subscale. Furthermore, consistent with clinical findings, the AI, NE, and NED subscales were strongly and positively correlated with each other, while PE was more feebly correlated with the other three dimensions.

Construct validity was also assessed in terms of gender-related differences. Indeed, males and females significantly differed in both clinical and non-clinical samples. As expected, girls scored higher in most ED dimensions, namely the AI, NE, and NED subscales, while EI was similar across gender. Moreover, gender differences were also detected in the relative distribution among groups, with ADHD exhibiting strong male prevalence and BSD with an even gender distribution. On the contrary, no age effect was found for any of the dimensions of dysregulation.

Correlations between RIPoSt and CBCL subscales further supported the concurrent validity of the new instrument. Indeed, the significant positive correlation between the AI, EI, NE, and NED subscales of the RIPoSt-40 and all syndromes and problems subscales and most DSM-oriented diagnostic category subscales of the CBCL-6/18, but especially their strongest and most significant correlations with the CBCL-DP, indicates that the four subscales and their combination can be used as an effective tool for studying ED in different clinical conditions. On the contrary, PE was limitedly correlated with the anxious/depressed, withdrawal/depressed, and affective problems subscales; thus, it seems to be only related with the affective dimensions of the CBCL, and notably, it did not exhibit a significant correlation with CBCL-DP.

Finally, when comorbidities were also taken into account, the AI, NE, and NED subscales presented a positive significant association with multiple anxiety disorder, which has been repeatedly found as a possible precursor of and frequently associated with BSD (47–49). Unexpectedly, disruptive behavior disorders did not show such an association, not even with the EI subscale. This result is in apparent contrast with previous findings from the available literature on the topic (22, 33) and would need further research. Indeed, disruptive behavior disorders are heterogeneous conditions, according to associated emotional features, in which ED, present in a strong minority of disruptive patients, may be specifically characterized by a deficit in emotional and behavioral self-control, with a greater risk of externalizing and aggressive behaviors (41).

This study should be considered preliminary, given some significant limitations: first of all, the lack of a formal standardization of the RIPoSt questionnaire in young people. Indeed, psychometric validation of multiple-item scales is considered to be an integral part, if not a crucial step, of data analysis in most substantive research studies (50). We largely based our study on the recent validation of the instrument in an adult sample of both clinical and non-clinical individuals (38), but future studies aimed at robustly validating and psychometrically assessing the RIPoSt in youth will be definitely required. Moreover, despite replicating common male-to-female ratio distributions in clinical samples of ADHD and BSD as ordinarily reported in literature (51) and correcting for gender whenever required in statistical comparisons, our samples significantly differed in terms of gender distribution. Future studies with more homogeneous distributions, or rather with larger proportions of the lacking sex, are warranted. Other limitations also include that we recruited modestly sized clinical samples and compared them with a school-based control group; nonetheless, we supposed this latter to be representative of the general population and applied strict exclusion criteria to prevent non-healthy controls to be recruited. Finally, we could not control for medication use and current interventions as potential confounding factors, which may affect our results, since full data were not available.

Despite these limitations, our study paved the way for future directions of research in clinical practice. Indeed, a thorough validation of the RIPoSt questionnaire along with an assessment of its psychometric properties is warranted. Our results also need to be further corroborated in larger samples. As pointed out before, the RIPoSt represents a potential clinical tool that may help in disentangling the complex relationship between ADHD and juvenile BSD for better diagnostic characterization and therapeutic indications. Future studies may further explore the longitudinal course of emotional dysregulation in these two partially overlapping disorders and assess the changes in their ED profile after psychopharmacological interventions. Moreover, the questionnaire may be used in the frame of evidence-based psychotherapeutic settings for psychopathological conditions characterized by ED to monitor the clinical course of its different dimensions and provide further evidence of effectiveness. Finally, the assessment of ED dimensions may be also useful in adolescents with conduct disorders, especially comorbid with ADHD, to further characterize the complex relationship between emotional regulation and executive functioning.

## Data Availability Statement

The raw data supporting the conclusions of this article will be made available by the authors, without undue reservation.

## Ethics Statement

The studies involving human participants were reviewed and approved by IRCCS Stella Maris Scientific Institute of Child Neurology and Psychiatry, Pisa, Italy. Written informed consent to participate in this study was provided by the participants' legal guardian/next of kin.

## Author Contributions

GM and AMi: conceptualization. GM, AMi, GS, and SP: methodology. CP, EV, AMo, FP, SB, NL, FD, and ARM: data collection and discussion on the first draft and conclusions. GS and SP: statistical analyses. GM, GS, AMi, and SP: writing first draft. All authors have read and agreed to the published version of the manuscript.

## Conflict of Interest

GM has received research grants from Lundbeck and Humana, was in an advisory board for Angelini, and has been speaker for Angelini, FB Health, Janssen, Lundbeck, and Otsuka. The remaining authors declare that the research was conducted in the absence of any commercial or financial relationships that could be construed as a potential conflict of interest.
